# Comparative effectiveness of implementation strategies for Accelerating Cervical Cancer Elimination through the integration of Screen-and-treat Services (ACCESS study): protocol for a cluster randomized hybrid type III trial in Nigeria

**DOI:** 10.1186/s13012-024-01349-9

**Published:** 2024-03-11

**Authors:** Babayemi O. Olakunde, Ijeoma U. Itanyi, John O. Olawepo, Lin Liu, Chinenye Bembir, Ngozi Idemili-Aronu, Nwamaka N. Lasebikan, Tonia C. Onyeka, Cyril C. Dim, Chibuike O. Chigbu, Echezona E. Ezeanolue, Gregory A. Aarons

**Affiliations:** 1 Department of Community Prevention and Care Services, National Agency for the Control of AIDS, Abuja, Nigeria; 2 Center for Translation and Implementation Research, University of Nigeria Nsukka, Enugu, Nigeria; 3 Department of Community Medicine, College of Medicine, University of Nigeria Nsukka, Enugu, Nigeria; 4 Department of Health Sciences, Northeastern University, Boston, MA USA; 5 Herbert Wertheim School of Public Health & Human Longevity Science, University of California San Diego, La Jolla, CA USA; 6 Department of Sociology and Anthropology, University of Nigeria Nsukka, Enugu, Nigeria; 7 Oncology Center, University of Nigeria Teaching Hospital, Enugu, Nigeria; 8 Department of Anaesthesia/Pain & Palliative Care Unit, College of Medicine, University of Nigeria Teaching Hospital, Ituku-Ozalla, Enugu, Nigeria; 9 Department of Obstetrics and Gynecology, College of Medicine, University of Nigeria Teaching Hospital, Ituku-Ozalla, Enugu, Nigeria; 10 HealthySunrise Foundation, Las Vegas, NV USA; 11 Department of Psychiatry, University of California San Diego, La Jolla, CA 92093-0812 USA; 12 UC San Diego ACTRI Dissemination and Implementation Science Center, La Jolla, CA USA; 13 Moores Cancer Center, University of California San Diego, La Jolla, CA 92093 USA

**Keywords:** Africa, Nigeria, Cervical cancer, Implementation science, Women living with HIV, EPIS framework, Exploration, Preparation, Implementation, and Sustainment framework, RE-AIM

## Abstract

**Background:**

Despite the increased risk of cervical cancer (CC) among women living with HIV (WLHIV), CC screening and treatment (CCST) rates remain low in Africa. The integration of CCST services into established HIV programs in Africa can improve CC prevention and control. However, the paucity of evidence on effective implementation strategies (IS) has limited the success of integration in many countries. In this study, we seek to identify effective IS to enhance the integration of CCST services into existing HIV programs in Nigeria.

**Methods:**

Our proposed study has formative and experimental activities across the four phases of the Exploration, Preparation, Implementation, and Sustainment (EPIS) framework. Through an implementation mapping conducted with stakeholders in the exploration phase, we identified a core package of IS (Core) and an enhanced package of IS (Core+) mostly selected from the Expert Recommendations for Implementing Change. In the preparation phase, we refined and tailored the Core and Core+ IS with the implementation resource teams for local appropriateness. In the implementation phase, we will conduct a cluster-randomized hybrid type III trial to assess the comparative effectiveness of Core versus Core+. HIV comprehensive treatment sites (*k* = 12) will be matched by region and randomized to Core or Core+ in the ratio of 1:1 stratified by region. In the sustainment phase, we will assess the sustainment of CCST at each site. The study outcomes will be assessed using RE-AIM: reach (screening rate), adoption (uptake of IS by study sites), IS fidelity (degree to which the IS occurred according to protocol), clinical intervention fidelity (delivery of CC screening, onsite treatment, and referral according to protocol), clinical effectiveness (posttreatment screen negative), and sustainment (continued integrated CCST service delivery). Additionally, we will descriptively explore potential mechanisms, including organizational readiness, implementation climate, CCST self-efficacy, and implementation intentions.

**Discussion:**

The assessment of IS to increase CCST rates is consistent with the global plan of eliminating CC as a public health threat by 2030. Our study will identify a set of evidence-based IS for low-income settings to integrate evidence-based CCST interventions into routine HIV care in order to improve the health and life expectancy of WLHIV.

**Trial registration:**

Prospectively registered on November 7, 2023, at ClinicalTrials.gov no. NCT06128304. https://classic.clinicaltrials.gov/ct2/show/study/NCT06128304

**Supplementary Information:**

The online version contains supplementary material available at 10.1186/s13012-024-01349-9.

Contributions to literature

- This study will be the first to test the effectiveness of tailored implementation strategies (IS) for cervical cancer screening and treatment (CCST) among women living with HIV in Nigeria.
- The study integrates two implementation science frameworks: The Exploration, Preparation, Implementation, and Sustainment (EPIS) Framework and Reach, Effectiveness, Adoption, Implementation, and Maintenance (RE-AIM) to guide implementation process, engage collaborators, and assess implementation outcomes.
- The ACCESS project advances a community-engaged collaborative approach to implementation strategy development and tailoring across diverse regions of an entire country for improving cancer control in low-and middle-income countries.
- The use of the implementation resource team approach engages collaborators through the EPIS process phases to refine and tailor implementation strategies for local appropriateness and facilitate the documentation of planned and ad hoc adaptations during the study.

## Background

Cervical cancer (CC) is the second most common cancer in women in Africa [1] and is a large contributor to cancer deaths in the region [2]. While age-standardized mortality trends have declined in high-income countries, mortality is rising in many low-income countries [3]. By 2030, CC will account for more than 106,000 deaths annually in Africa, an increase of 38% from the estimated 77,000 deaths in 2020 [4]. Compared with HIV-negative women, women living with HIV (WLHIV) are six times more likely to develop CC [5], and are at increased risk of mortality from CC [6–9]. About 25% of CC cases in Africa are diagnosed in WLHIV, and 21% are attributable to HIV [10]. With a 5-year prevalence of 22,500 cases [11] and 960,000 WLHIV [12], Nigeria has one of the largest burdens of CC and HIV in Africa.

CC is completely preventable. It is also curable if diagnosed and treated early. The availability of effective interventions, including human papillomavirus (HPV) vaccination and screening and treatment of precancerous lesions of CC [13], underpins the current global effort to eliminate CC as a public health problem [14]. The World Health Organization (WHO) has set measurable global targets of 90–70–90 (90% of girls fully vaccinated with HPV vaccine by 15 years of age, 70% of women screened using a high-performance test, and 90% of women identified with preinvasive cancer and invasive CC treated) to prevent and treat CC by 2030 [15].

CC screening and treatment (CCST) is an important evidence-based intervention for CC prevention and control. However, many African countries have not been able to implement and sustain organized national CC screening programs [16]. Although the availability of low-cost screening methods such as visual inspection with acetic acid (VIA) or Lugol’s iodine (VILI) for CC screening [17, 18] offers opportunities to improve early detection of CC in Africa, screening rates among women, including WLHIV, remain low [19]. In Nigeria, despite the recommendations of routine CC screening of WLHIV [20], studies have found screening coverage of < 10% among WLHIV [21–24]. CC screening in Nigeria is affected by several factors such as the unavailability of screening services, screening not being offered by healthcare providers, and poor awareness of the disease and screening services [25–28]. Efforts to improve the control of CC and other cancers in Nigeria have included the designation of one federal tertiary hospital in each of the country’s six geopolitical regions as oncology centers of excellence, where comprehensive cancer screening, diagnosis, and treatment can be accessed.

In low- and middle-income countries (LMIC) with a high burden of HIV, the integration of CCST services into the established HIV programs can improve CC prevention and control [29–31]. Investment in the HIV response has improved access to antiretroviral therapy (ART) in many African countries [32, 33]. For example, the US President’s Emergency Plan for AIDS Relief (PEPFAR) HIV care program in Nigeria has invested more than US $6 billion in Nigeria’s national HIV response [34]. PEPFAR has contributed to health systems strengthening through human capacity development, establishing electronic health management information systems, and providing state-of-the-art laboratories to adequately respond to the HIV epidemic and other diseases in Nigeria [35]. These existing infrastructures for HIV can be leveraged to deliver CCST services. Depending on the availability of physical and human resources, the integration of CCST services into HIV services may be within the same clinic or through referral [36].

While evidence suggests that the integration of CCST into HIV services is feasible and acceptable [36–40], it has not been widely implemented at scale or yielded the desired outcomes in many low-resource, high-burden countries, including Nigeria [15]. The gap in knowledge of effective implementation strategies (IS) to enhance integration of CCST into HIV services has limited its success and scale-up [30, 41]. Findings from previous reports [40, 42, 43] indicate that effective integration of CCST for WLHIV will require tailored IS that will simultaneously address multilevel barriers in the outer context of the country health systems and the inner organizational contexts of HIV clinics [44, 45].

Accordingly, guided by the Exploration, Preparation, Implementation, and Sustainment (EPIS) framework [44, 46], we will determine the barriers to and facilitators of CCST implementation and sustainment and identify and test promising implementation strategies to enhance the integration of CCST into existing HIV programs in Nigeria. EPIS is both a process and determinant framework that describes four phases of the implementation process to guide researchers and implementers in the process. We utilized the EPIS process phases to stage the study while also invoking consideration of determinants and mechanisms (i.e., barriers and facilitators). Determinants are identified in the outer system context (e.g., cultural and populations variation in regions), inner organizational context (i.e., clinic operations), innovation factors (i.e., characteristics of CCST), and bridging factors that connect outer and inner contexts [47]. Importantly, EPIS also recognizes the importance of relationships, interconnections, and linkages among and between actors in outer and inner contexts. Our implementation and clinical outcomes are informed by the Reach, Effectiveness, Adoption, Implementation, and Maintenance (RE-AIM) framework. As part of study development, the EPIS exploration phase was completed in which we collaborated with stakeholders in Nigeria who participated in the following: (1) identifying and ranking implementation barriers and facilitators and (2) matching and ranking IS to the identified determinants [48]. As shown in Table 1, the results from the implementation mapping and the refinement are a set of a core package of IS (Core) and an enhanced package of IS (Core+).

**Table 1:**
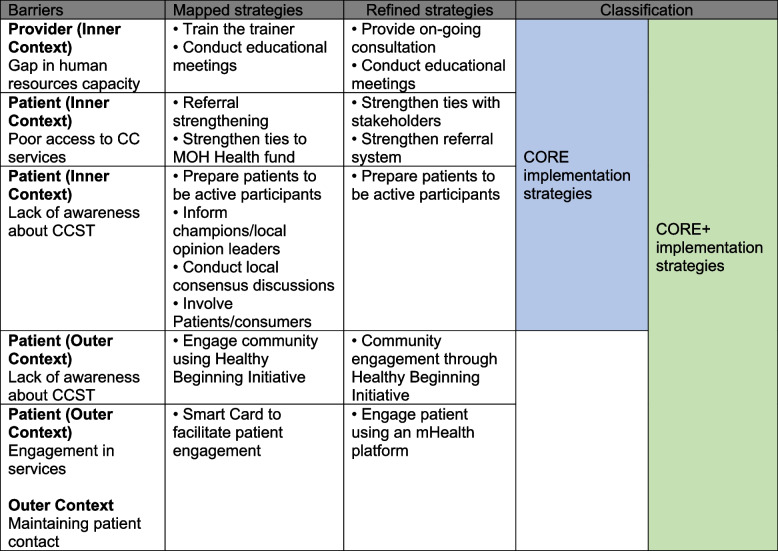
Barriers and strategies from stakeholder implementation mapping and refinement

Our specific aims are as follows:

- Aim 1: Refine strategies to integrate CCST of cervical precancer within existing comprehensive HIV treatment programs.
- Aim 2: Determine the comparative effectiveness of the Core vs Core+ implementation strategies on CCST*.*
- Aim 3: Assess the sustainment of the integration of CCST into comprehensive HIV treatment programs.

## Methods

### Overview of study preparation and design

This is a cluster randomized trial using hybrid type III implementation-effectiveness study type [49] to assess the comparative effectiveness of a set of Core and enhanced (i.e., Core+) implementation strategies on the implementation and sustainment of cervical cancer screening and treatment for WHLIV. The hybrid type III design focuses primarily on testing IS effectiveness while observing clinical effectiveness outcomes [49]. HIV comprehensive treatment centers (*k* = 12) will be matched by region and randomized 1:1 to Core or Core+ strategies. We will assess both implementation and clinical outcomes and explore potential mechanisms that affect the study outcomes, including organizational readiness, implementation climate, CCST self-efficacy, and implementation intentions. The study is guided by two implementation science frameworks — EPIS and RE-AIM.

### Exploration, preparation, implementation, and sustainment (EPIS) framework

**Fig. 1:**
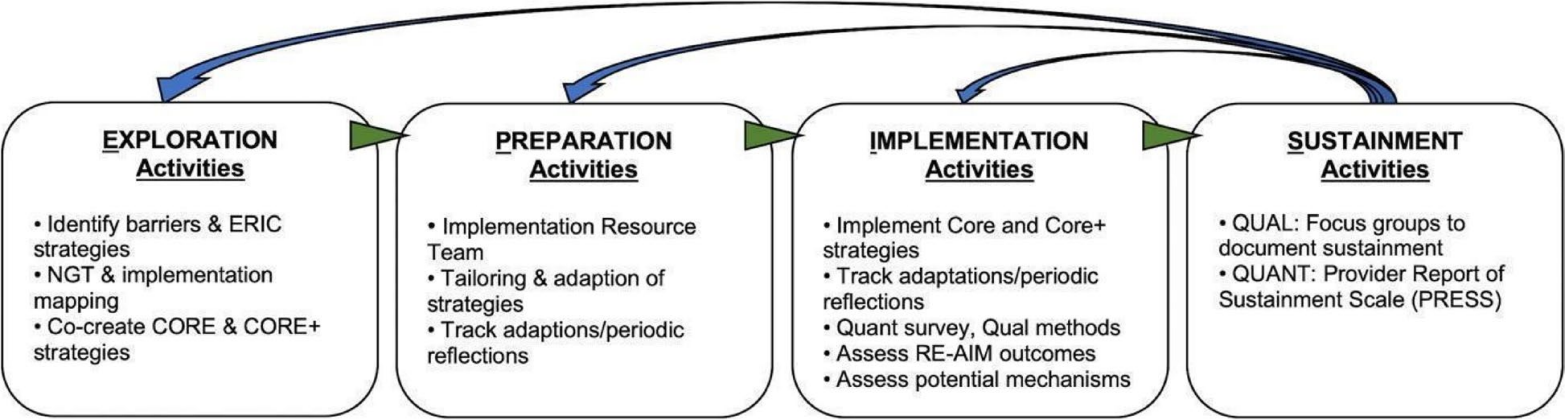
EPIS framework activities and outer/inner contexts

EPIS was selected because it is useful in guiding implementation in different settings including low-income countries [44, 46, 50]. Figure 1 shows the activities within each phase.

Prior to grant submission and funding, we completed the *exploration phase* [48] where we used survey and modified nominal group techniques (NGT) [51] to conduct a rapid and facilitated implementation mapping [52] exercise. Collaborators identified and ranked barriers and identified, selected, and ranked implementation strategies starting with the Expert Recommendations for Implementing Change (ERIC) [53] and other identified additional strategies. The final set of strategies were selected to address the barriers to integration of CCST into HIV treatment programs to form the Core IS. Stakeholders identified two additional strategies to be included in the Core+ IS condition [48].

In the *preparation phase*, we refined the IS and convened implementation resource team (IRT) [54] meetings to further reflect on the refined Core and Core+ IS fit with local context. Implementation resource teams were first conceptualized and tested in the dynamic adaptation process model and the Interagency Collaborative Team model for scaling up evidence-based practices [54, 55]. The IRT for this study is a community-engaged co-creation collaborative comprising the research team and the doctors and nurses from the study sites. Twelve site research coordinators (SRCs) were also recruited and trained during this phase.

The process of refinement of our proposed implementation strategies occurred in two phases. In the first phase, the research team developed an IS table which included the identified implementation determinants (barriers/facilitators), the initially proposed IS for each determinant, a definition of each IS, the population targeted by the IS, the proposed actions for the IS, and the responsible person(s) for these proposed actions. For each IS, we discussed its feasibility and importance in addressing the implementation determinant based on the IRT’s current knowledge of the context in the implementation sites. For each IS that IRT identified as not being the most feasible or important to address the implementation determinant, we selected another IS from the same category in the ERIC grouping of implementation strategies [53], and we included some strategies not included in ERIC. The refined strategies are shown in Table 1.

In the second phase, 12 doctors and 12 nurses, 1 from each study site, participated in 2 focus-group discussions (FGDs) to further refine the IS. Each FGD was facilitated by a member of the research team with experience in qualitative data collection, using a semi-structured FGD guide, while two members of the research team took notes. Written informed consent was obtained from all the participants after reading the information about the study and the FGDs and given the opportunity to ask questions. Verbal consent was also obtained from the participants for the audio recording of the FGDs. The participants were presented with the refined implementation strategies table developed in the first phase and were asked to contribute to further refinement of the table, particularly tailoring the target population, planned actions, actor, dose, and mode of delivery. They were also asked to state if any of the IS or their activities would not be feasible at their sites. For any IS where the participants had different views about tailoring, discussions were held, and a consensus was reached. Each FGD lasted for about 2 h. Details of the further refined strategies are shown in Table 2. All of this work was in preparation for the cluster randomized trial to be described in the next section.

**Table 2:** Implementation strategies

Arm	Strategy	Definition	Actor	Action	Target	Dose	Delivery
Core and Core+	Provide ongoing consultation	Provide ongoing support to address specific site CCST training needs	Practice-based Research Network Site clinical team	Conduct real-time support and periodic training sessions on CCST through the review of cases captured on AVIVA app (a mobile app that allows images (native cervix, acetic acid- and Lugol iodine-stained cervix) to be uploaded for expert’s review) [59]	Site clinical team	Clinic days (real time) Quarterly (periodic sessions)	Virtual
Core and Core+	Conduct educational meetings	Hold meetings among healthcare providers to improve CCST service provision	Site clinical team	Conduct site clinical review meetings on CCST cases, number screened and treated within review period and any other quality improvement issue	Site clinical team	Monthly	In-person
Core and Core+	Strengthen ties with stakeholders	Hold meetings with policymakers and donor partners to facilitate availability of commodities and equipment for CCST	Research team	Conduct stakeholder meetings	Ministry of Health PEPFAR IPs Site clinical teams Site medical directors	Quarterly	Virtual
Core and Core+	Strengthen referral system	Facilitate referral and easy navigation at referral centers to ensure referred WLHIV with suspected cancer complete referral	Site clinical team	Identify and prepare individuals as referral coordinators to track and support easy navigation at referral centers	WLHIV	Continuous	In-person
Core and Core+	Prepare patients to be active participants	Prepare patients to be active participants in their care	Site clinical team	Distribute flyers/leaflets/flip charts on CCST	WLHIV Partners of WLHIV	Continuous	In-person
Core+	Community engagement through Healthy Beginning Initiative (HBI)	Engage communities of WLHIV through a congregational-based approach [60–62] to educate and create awareness about CC and CCST	Site clinical team Community health team	Conduct HBI incorporating CC and CCST education at HIV support group meetings	PLHIV	Quarterly	In-person
Core+	Engage patient using an mHealth platform	Use patient-held smartcard [63, 64] linked to an mHealth platform to record CCST services and track and send appointment reminders to WLHIV	Research team/clinical team	Scan patients’ smartcards and capture CCST information at every study visit Send appointment reminders to patients	WLHIV	Continuous	In person

*CC* cervical cancer, *CCST* cervical cancer screening and testing, *IP* implementing partners, *PLHIV* people living with HIV, *WLHIV* women living with HIV

In the *implementation phase*, we will implement the Core and Core+ IS and test their comparative effectiveness on select implementation outcomes while collecting data on potential mechanisms of effects and clinical effectiveness outcomes. We will also catalog any ad hoc adaptations and contextual factors affecting implementation, services, and clinical outcomes with a focus on sustainment.

In the *sustainment phase*, we will assess sustainment of CCST and IS at each site and provide summaries to stakeholders regarding the planned and ad hoc adaptations that occurred during implementation.

### Reach, Effectiveness, Adoption, Implementation, and Maintenance (RE-AIM)

We selected RE-AIM as our evaluation framework for assessing individual- and setting-level outcomes important to program impact and sustainment [56, 57]. As shown in Table 3, we address relevant RE-AIM constructs: Reach, Effectiveness, Adoption, Implementation, and Maintenance (also known as Sustainment) [56, 57]. In operationalizing these five dimensions, we adapted the metrics recommended by the Integrative Systems Praxis for Implementation Research (INSPIRE) model [58]. We defined the constructs as follows: reach (screening rate); clinical effectiveness (posttreatment screen negative); adoption (uptake of IS by study sites); IS fidelity (degree to which the IS occurred according to protocol); implementation clinical intervention fidelity (delivery of CC screening, onsite treatment, and referral according to protocol); and maintenance (or sustainment) (the degree to which integration of CCST continued).

**Table 3:** Measures by EPIS phase and study aims

Aim	Construct	Measurement	Data source
EPIS preparation and implementation phases			
2	Reach (EPIS inner context)	•% WLHIV who had CC screening	Health records Participant survey
2	Effectiveness (EPIS inner context)	•% WLHIV treated for preinvasive cancer who had negative posttreatment follow-up screening	Health records Participant survey
2	Adoption	•% study sites that used AVIVA app to capture CC cases for real-time support •% study sites that participated in the periodic review training sessions on CCST •% study sites that initiated monthly educational meetings •% study sites that participated in the quarterly stakeholder meetings •% study sites that identified a referral coordinator to follow through with referred WLHIV with suspected cancer and ineligible lesions for treatment •% study sites that distributed flyers/leaflets/flipcharts on CCST in clinics •% study sites (Core+) that conducted HBI at support group meetings •% study sites (Core+) that captured CCST data of recruited WLHIV on smart card •% study sites (Core+) that send appointment reminders to patients using the mHealth platform	Survey Documented records
2	Implementation strategy fidelity	•(QUANT) 7 items (for Core) and 11 items (for Core+) with subscales, degree that components of each strategy were implemented in terms of adherence, exposure, quality, and engagement	Survey Documented records Periodic reflection
2	CCST intervention fidelity (EPIS inner context)	•% WLHIV with preinvasive cancer eligible for treatment who had same-day treatment •% WLHIV treated for preinvasive cancer who had posttreatment follow-up screening at 12 months •% WLHIV with suspected cancer or lesions ineligible for treatment referred	Health records Participant survey
2	Organizational readiness for CCST	•(QUANT) organizational readiness for implementation (ORIC)	Provider survey
2	Implementation climate (EPIS inner context)	•(QUANT) six items with three subscales, degree that CCST is expected, supported, and rewarded	Provider survey

#### Site selection

**Fig. 2:**
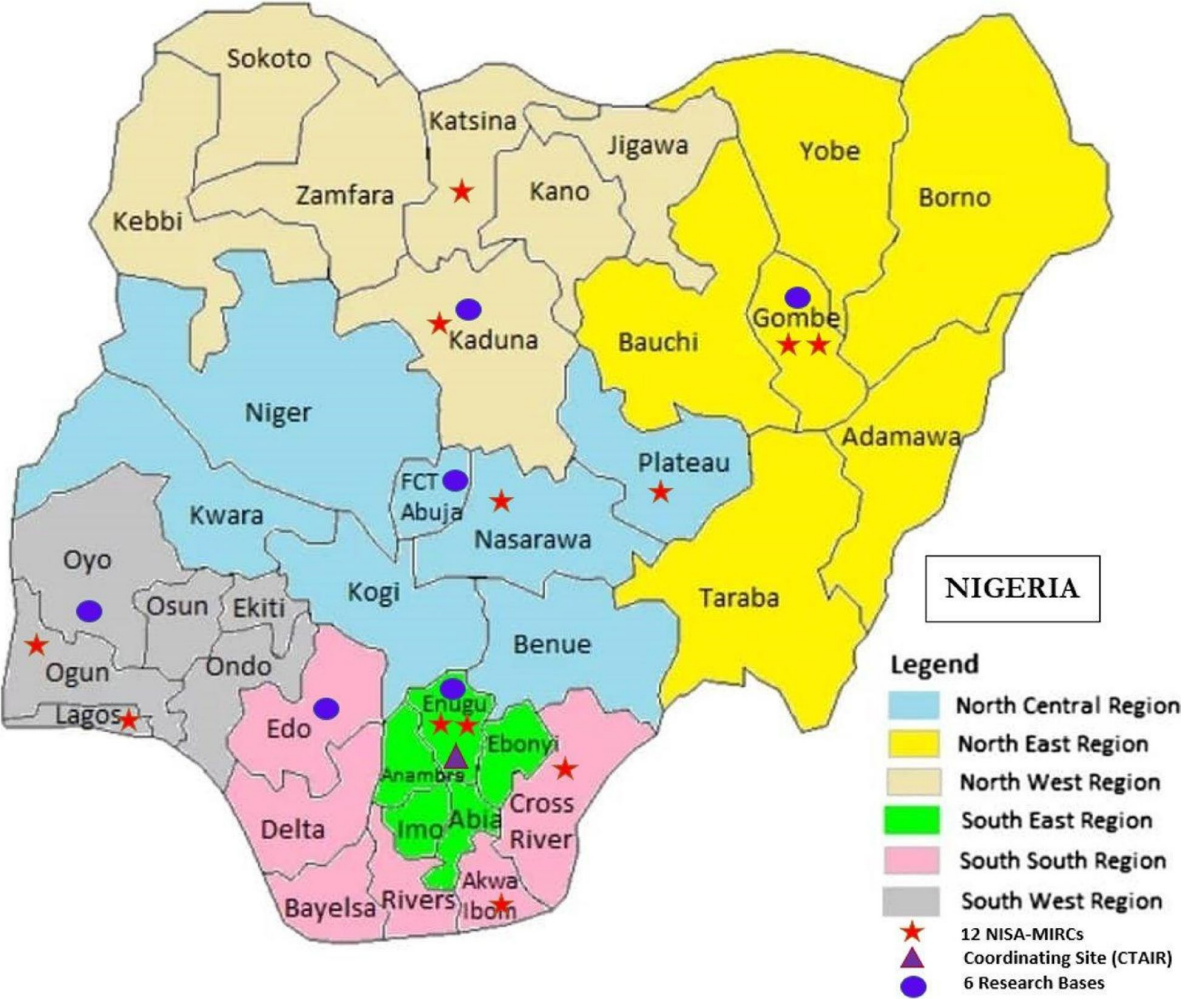
12 NISA-MIRCs selected for ACCESS study

The study will leverage the 21 comprehensive HIV treatment sites across the six geopolitical zones of Nigeria that have been designated as Nigeria Implementation Science Alliance (NISA) Model Innovation and Research Centers (NISA-MIRC) [65]. The NISA-MIRC sites are part of the ICON-3 Practice-based Research Network which is made up of the 21 NISA-MIRCs and the 6 Regional Centers of Excellence. The NISA-MIRCs have over 60,000 WLHIV in care who are eligible for enrollment into clinical trials and other implementation research studies [65]. Our proposed study will be anchored at 12 sites (see Figure 2 and Additional file 1) from the 21 NISA-MIRCs. The criteria for selection were based on the following: (1) geographical spread (two sites per region), (2) highest proportion of consented WLHIV for enrolment into clinical trials and other implementation research studies, and (3) match of the same type of site per region (secondary or tertiary).

#### Randomization

**Fig. 3:**
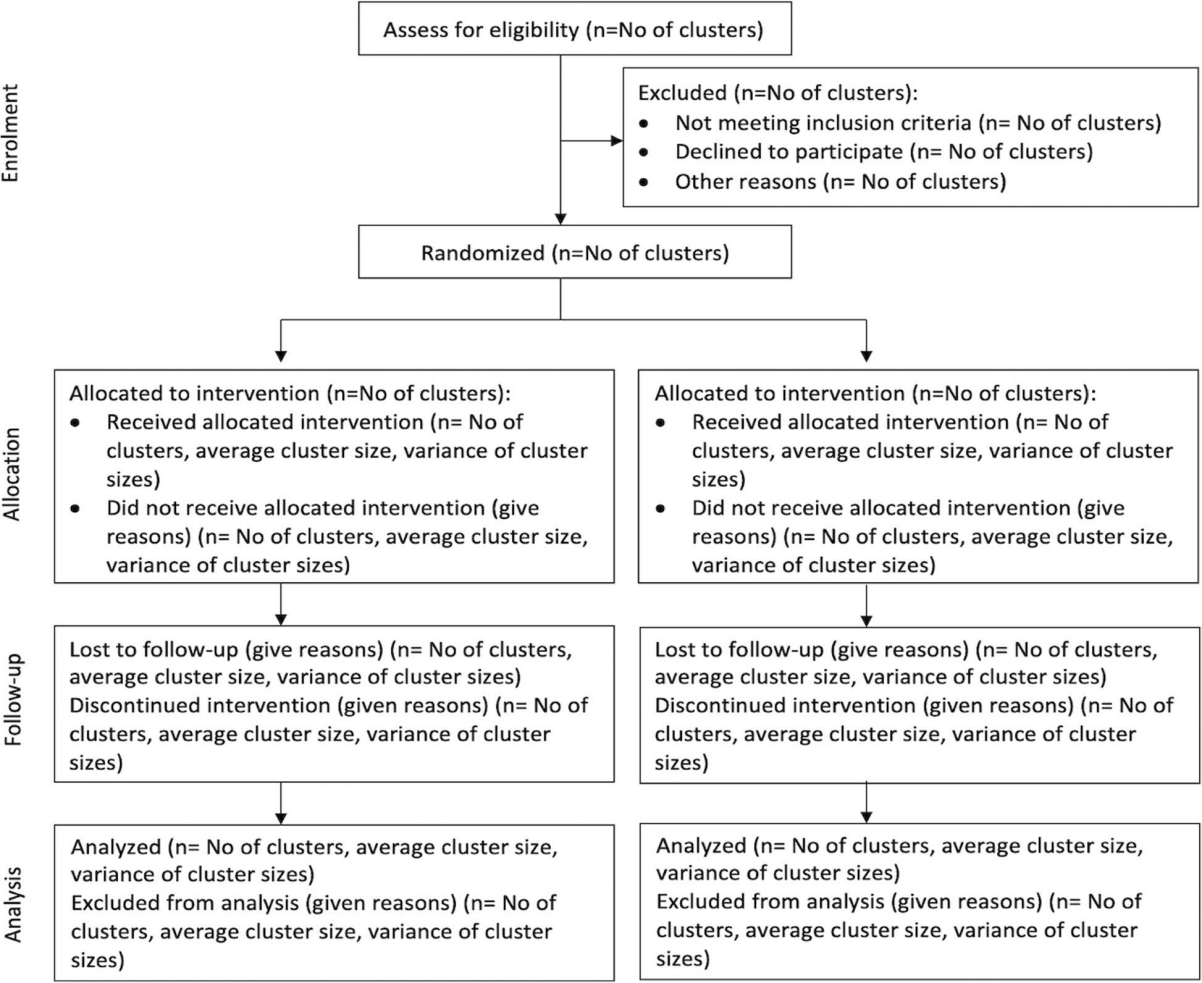
CONSORT flow diagram

The unit of randomization will be the sites. Each site represents a cluster in the randomization representing the clinic site and the WLHIV receiving treatment at the site. We will conduct simple random allocation with the sites randomized to either the Core or Core+ IS. There are six geographic regions in Nigeria, and sites will first be matched by region, and then each site in a matched pair will be randomized to Core or Core+ IS. A random number generator will be used to assign the sites. A flow diagram for the randomization and intervention allocation is shown in Figure 3. The healthcare workers and the patients will be blinded to whether their sites are allocated to the core package of IS or an enhanced package of IS.

### Methods for each study aim

- *Aim 1: To refine strategies to integrate cervical cancer screening and treatment of cervical precancers within existing comprehensive HIV treatment programs*. This has been described above in the EPIS preparation phase, and the findings are included in Tables 1 and 2.
- *Aim 2: To determine the comparative effectiveness of the Core vs Core+ Implementation Strategies on CSST*

### Study population

To be eligible for enrolment into the trial, a woman must meet all the following criteria:

- Be between 25 and 63 years
- Have been diagnosed with HIV
- Enrolled into HIV care in the sites

WLHIV will not be eligible to be enrolled into the trial if they have been previously diagnosed with preinvasive or invasive CC, received CC screening at any time within the previous 12 months, or are not willing to participate in the study procedures.

### Recruitment and retention

The medical records team will provide a list of potentially eligible WLHIV based on the eligibility criteria. The eligible WLHIV will be approached by the SRCs for recruitment into the study. For those who indicate interest, the SRCs will provide more details about the study, after which signed informed consent will be obtained electronically using REDCap. The consent forms will be read to all the participants in the English language. When there is a language barrier, the multilingual SRCs will interact with the potential participants in their local dialect. At enrollment, appointments for their next visit (data collection) will be scheduled.

### Data collection

Sociodemographic and baseline data will be collected from the enrolled participants who will also be followed up to assess the outcomes of interest using structured follow-up questionnaires in addition to their electronic medical records. Data on potential determinants and mechanisms will be obtained from the healthcare providers, using the following validated instruments (see Additional file 2).

#### Organizational Readiness for Implementing Change (ORIC)

The ORIC assesses organizational readiness through two subscales, change commitment, and change valence. The ORIC has established high inter-item consistency and inter-rater reliability for both subscales [66]**.**

#### Implementation climate measure (ICM)

The six-item ICM has three subscales that assess perceptions of the degree to which a specific evidence-based practice (EBP) or innovation is expected, supported, and rewarded (i.e., recognition) in a clinic or organization [67]. The ICM has established strong psychometric properties.

#### Measure of innovation-specific implementation intentions (MISII)

The MISII is a three-item pragmatic measure of direct service providers’ (e.g., physicians, nurses, providers) intentions to engage with and use a specific innovation such as CCST [68]. The MISII has strong psychometric properties.

#### Self-Efficacy/EBP Beliefs Scale

This scale assesses the perceived value of the EBP and self-efficacy regarding the ability to implement the EBP. We will use the seven items that directly assess self-efficacy and we will tailor them to refer to CCST [69]**.**

#### Outcome measures

The measures for the implementation and clinical outcomes and the potential mechanisms are summarized in Table 3.

### Sample size and power calculation

We examine the sample size for the primary implementation outcome of “reach,” indicated by the CC screening rate in WLHIV. Based on preliminary data from previous studies, we assumed a conservative screen rate of 14% in the core implementation strategy group [70]. We plan to enroll 12 sites (6 sites per intervention group). Hade et al. [71] reported the range of intraclass correlation (ICC) fall in 0.02–0.07 in the group-randomized breast and CC screening studies. We performed a sensitivity analysis by assuming a range of ICC of 0.02–0.07 for site cluster effect on the outcome and a feasible sample size of 200 subjects per site. We estimated that we could achieve at least 80% power for ICC of 0.02–0.06 (84% for *ICC* = 0.06 and > 99.9% for *ICC* = 0.02) to detect a minimum improvement of 20 percentage points in screening rate (that is, 34% screening rate) in Core+ group using a two-sided type I error of 0.05, and we could still achieve 78.6% power for *ICC* = 0.07. The 34% screening rate is achievable and a conservative estimate for the Core+ group based on our prior related work [61]. The power analysis was conducted using the statistical software R package *cluster Power* [72].

### Data analysis

Preliminary analyses will begin with an examination of the distribution of variables to assess and describe their characteristics (means, standard deviations, quartiles, ranges, frequencies, and percentages) for overall and for Core and Core+ groups separately and to allow assessment of randomization. Randomization will be tested by performing a series of Wilcoxon rank-sum tests for continuous variables and chi-square (or Fisher’s exact) tests for categorical variables to compare the groups on baseline demographic and clinical variables. Variables on which the groups differ initially will be explored as covariates in subsequent analysis. Intent-to-treat analysis will be conducted as the primary analysis. All estimates (point estimates and 95% confidence intervals) for the study outcomes will be adjusted by the cluster effect of clinics as described below.

The screening rate (primary outcome) will be summarized by frequency and proportion. A generalized linear mixed-effect model (GLMM) [73] with a binomial link will be used to examine the difference in screening rate (reach outcome) between Core and Core+. A random intercept will be included in the model to account for the cluster effect of the site. Multivariable random effects models will be used to examine the impact of covariates on estimated intervention effects. The GLMM will be also used to compare secondary outcome measures related to adoption, implementation strategies fidelity, and clinical intervention fidelity between two intervention groups, with a binomial link for a binary outcome and a Gaussian link for a continuous outcome. The ICM, MISII, and Self-Efficacy/EBP Beliefs Scale data collected from providers will also be analyzed similarly using the GLMM.

#### Covariate prescreening and variable selection procedure

In the multivariable model described above, adjustments are typically made to correct for baseline imbalances between groups and to adjust for variables known to influence the outcome independent of the intervention. Baseline characteristics will be pre-screened and assessed for an imbalance between the two intervention groups (Wilcoxon rank-sum test or Fisher’s exact test) and their association with the outcome (univariable mixed-effects model). Only those variables found to be moderately associated (*p* < 0.15) with the outcome or imbalanced (*p* < 0.10) between groups will be considered as potential covariates in the initial multivariable model. Backward elimination of insignificant variables will be used to select the main effects in the final model; all covariates that are significant at *p* < 0.10 will be kept in the final model.

#### Missing data

In the case of missing data, missing patterns will be assessed by comparing patient characteristics between patients with and without missing data. Appropriate data analytic techniques will be used for analysis, which may include deletion, imputation, and inclusion of an indicator of missing values.

- *Aim 3: To assess the sustainment of the integration of CCST into HIV programs*

### Study population

This will include healthcare providers (doctors and nurses) that provided CCST for WLHIV and healthcare administrators (medical directors) in the NISA-MIRCs study sites.

### Recruitment

Healthcare providers/administrators will be invited via letters and emails to participate in a survey and FGD.

### Data collection

Quantitative data will be collected from the healthcare providers/administrators using the Provider REport of Sustainment Scale (PRESS) [74]. The PRESS is a brief and pragmatic three-item measure of sustainment that can be used across different EBP, provider types, and settings. The PRESS captures clinic staff’s report of their clinic, team, or agency’s continued use of an EBP or innovation in practice. The PRESS has excellent psychometric characteristics. Items are measured on a 5-point Likert scale ranging from (0 [not at all] to 4 [to a very great extent]. Qualitative data regarding sustainment will be collected using FGD with doctors, nurses, and administrators in each of the sites.

### Sample size

About 36–48 health care providers (3–4 per site) will be surveyed using PRESS. We will conduct 2 FGDs (Core and Core+) with 12 participants per group.

### Data analysis

We will analyze data using a mixed-methods approach. From the quantitative data, we will classify sustainment status according to Wiltsey Stirman and colleague’s recommendations [75] and as used by Aarons and colleagues [76]. For the mixed-methods integration, we will use a QUAN+QUAL approach in which data are gathered and given equal weight [77]. We will triangulate qualitative and quantitative data to examine convergence, expansion, and complementarity when developing overall interpretations and conclusions [77–83]. We will create matrices to identify and summarize convergences and divergences in analyses of all data sources integrating results into a comprehensive picture [80]. We will first consider each type of analysis on its own terms and how they differ or converge [79, 84] by linking qualitative and quantitative databases and embedding one within the other so that each has a supportive function to play.

### Study procedures

**Fig. 4:**
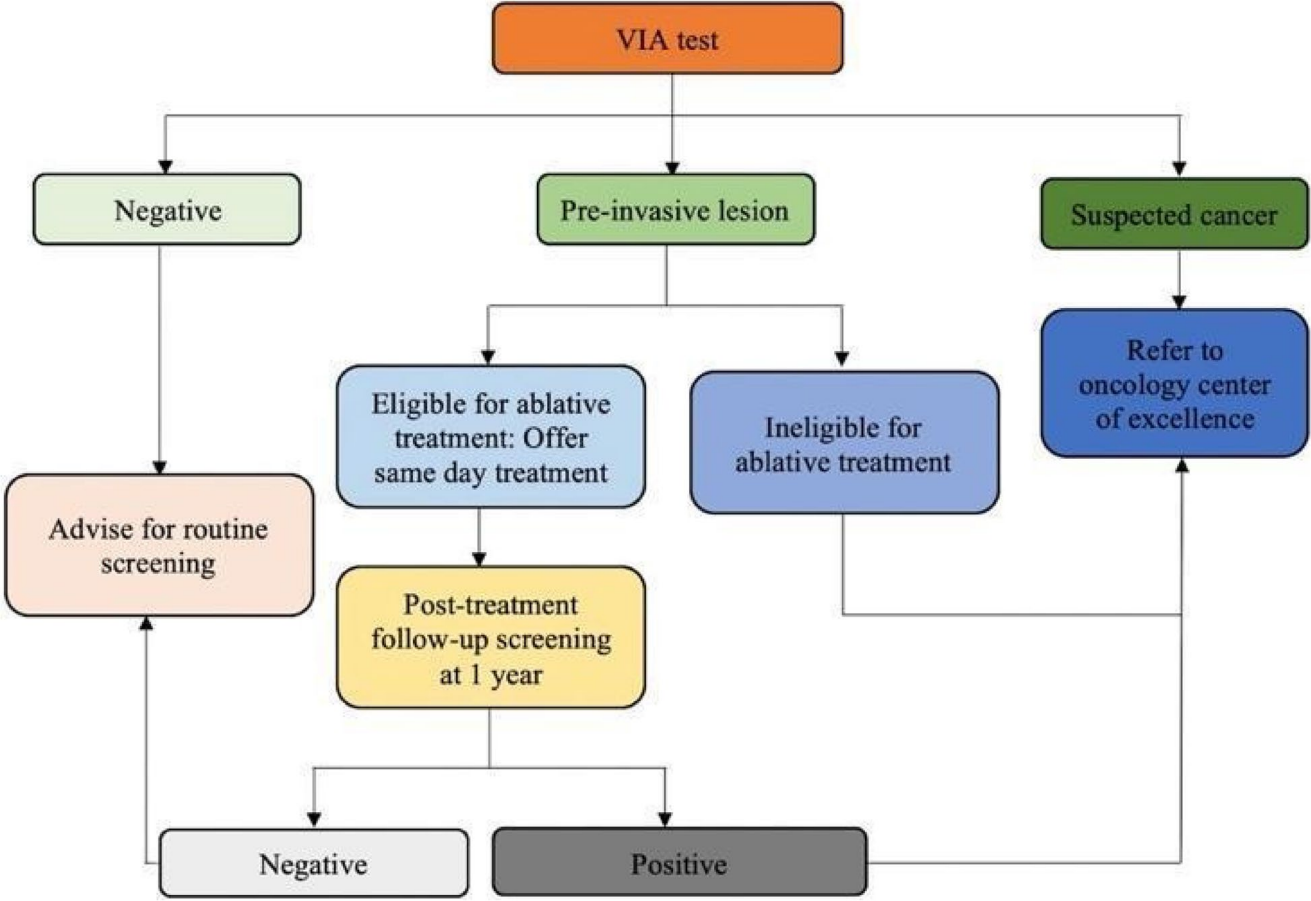
Algorithm for VIA-based screening, treatment, and referral

Due to the available infrastructure in the 12 study sites, CC screening in this study will be performed using VIA. In line with the standard of care in the sites, the decision to treat preinvasive cancer lesions will be based on the result of the screening test only, and not on a histologically confirmed diagnosis (screen and treat approach). Treatment of preinvasive cancer lesions will be through ablative therapy. Participants with preinvasive lesions that are not eligible for ablative treatment, or with suspected cancer lesions, will be referred to one of the regional oncology centers of excellence (Figure 4).

### Schedule of measurement

**Fig. 5:**
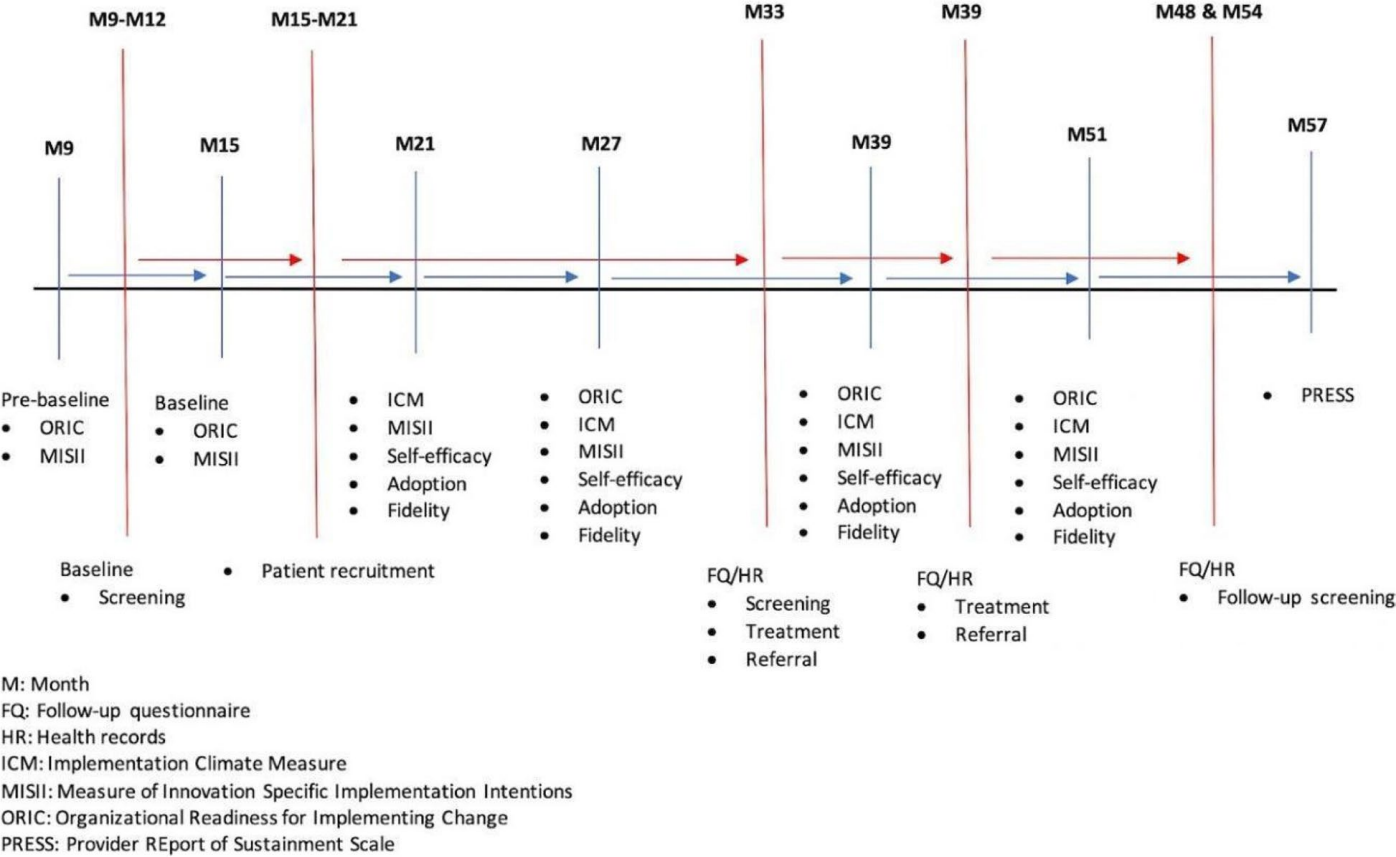
Study assessment

We will collect baseline data on the potential mechanisms (ORIC and MISII) and CC screening rates among WLHIV in the 12 facilities (Figure 5). In aim 2, each participant will be assessed at 12 months after enrollment for the receipt of CC screening (study M33). Treatment of precancerous lesions and referral of suspected cancer or lesions ineligible for treatment will be assessed at 12 and 18 months after enrollment (study M33 and M39). Posttreatment follow-up screening will be assessed at 27 and 33 months after recruitment (study M48 & M54). Data on specific potential mechanisms will be collected from the healthcare providers after recruitment and then 6 months, 12 months, and annually.

#### Tracking adaptations

We will use quarterly “periodic reflections” [85] with the entire investigative team to consider any ad hoc adaptations that occurred at any of the sites. We will adapt periodic reflection focus-group guides for the discussion around what adaptations may have occurred, why they occurred, at what system or organizational level, who was involved, and the time frame. We will catalog adaptations and place them within the EPIS framework identifying the phase when they occurred and whether they arose from or impacted EPIS determinants or mechanisms in outer context, inner context, bridging factors, or the innovation itself (i.e., CCST). We will also place adaptations within the FRAME-IS model for cataloging and reporting adaptations [86, 87].

#### Data handling

We will manage study data using REDCap (Research Electronic Data Capture; http://project-redcap.org/). REDCap provides user-friendly secure web-based case report forms and real-time data entry. We will use password-protected and encrypted tablets, laptops, and external drives that are institutionally certified and issued for data storage. Study data will be shared with only principal investigator-approved study staff via secure file transfer platforms.

## Discussion

In this study, we will conduct a cluster randomized, hybrid type III trial among comprehensive HIV treatment centers in Nigeria (*k* = 12) for WLHIV (*N* = 2400) to assess the comparative effectiveness of an enhanced package of IS (Core+) versus a core package of IS (Core) on the integration of CCST into HIV programs. Upon conclusion, our study will identify a set of evidence-based IS for low-income settings to integrate CCST interventions into routine HIV care to improve the health and life expectancy of WLHIV.

Meeting the WHO targets of ending CC requires countries to identify innovative implementation strategies that address contextual barriers to integration [14]. Several implementation strategies such as training of providers, community outreach, educational materials, changing service sites, task shifting, ongoing consultation, patient reminder systems, and audit-feedback mechanisms have been used in improving cervical cancer prevention in sub-Saharan Africa [41]. However, most of the studies reporting these IS did not evaluate their effectiveness or report implementation-specific outcomes [41].

To our knowledge, our study will be the first to test the effectiveness of tailored IS on the implementation of CCST among WLHIV in Nigeria. The use of hybrid type III trial design will allow our study to gather reliable evidence on implementation and clinical effectiveness outcomes. Our approach of conducting a multicenter study comprising 12 sites across the six geopolitical regions of Nigeria will ensure the representativeness of findings. Also, leveraging the PEPFAR-supported programs in Nigeria and our established partnerships will increase the likelihood of successful study implementation and service sustainment. The use of IRT as a part of the EPIS process can serve as a stakeholder engagement approach as it has been utilized in other studies [54]. We have engaged the IRT to refine and tailor the IS for local appropriateness. They will also facilitate the documentation of planned and ad hoc adaptations during the study.

Nonetheless, there are potential limitations to this study. The inadequate number of fully equipped laboratories, and the current high cost of HPV DNA tests, limits the implementation of WHO-recommended HPV screen, triage, and treat (HPV-STT) approach for WLHIV [88]. However, if the landscape changes, we will work to incorporate HPV testing instead of VIA/VILI. The 12 NISA-MIRCs sites selected for this study are secondary health facilities; thus, our findings may not be applicable to primary and tertiary healthcare facilities due to varying inner contexts.

## Conclusion

There has been a significant increase in the uptake of ART among WLHIV in Africa; however, the coverage of CCST among WLHIV who are at increased risk of CC remains low. Efforts to successfully leverage the available infrastructure for HIV care and treatment programs in low-income countries for integrated service delivery have been limited by the paucity of evidence on effective IS. With this cluster randomized, hybrid type III trial design in Nigeria, we will test and identify effective IS to integrate CCST services into routine HIV care. Results from this study could inform better implementation of CC secondary prevention intervention to improve survival among WLHIV in resource-limited settings.

### Supplementary Information

**Additional file 1.** Description of study facilities.

**Additional file 2.** Measurement Instruments.

**Additional file 3.**

**Additional file 4.**

## Data Availability

Not applicable.

## Abbreviations

CC: Cervical cancer
CCST: Cervical cancer screening and treatment
EPIS: Exploration, Preparation, Implementation, and Sustainment framework
IRT: Implementation resource team
IS: Implementation strategy
NISA: Nigeria Implementation Science Alliance
PEPFAR: US President’s Emergency Plan for AIDS Relief
SRC: Site research coordinators
WLHIV: Women living with HIV
