# Supplementary material for: Comparative effectiveness of implementation strategies for Accelerating Cervical Cancer Elimination through the integration of Screen-and-treat Services (ACCESS study): protocol for a cluster randomized hybrid type III trial in Nigeria
Source: Implement Sci. 2024 Mar 11;19:25. doi: 10.1186/s13012-024-01349-9 (PMC10926605; doi:10.1186/s13012-024-01349-9)
Supplement: Supplementary file 1 — Additional file 1. Description of study facilities. [file 13012_2024_1349_MOESM1_ESM.pdf]

Additional file 1: Description of study facilities

SN	Name of Facility	Location	Facility Type	Geopolitical zone
1	Annunciation Specialist Hospital, Emene, Enugu State	Urban	Secondary	South East
2	Mother of Christ Specialist Hospital, Ogui, Enugu	Urban	Secondary	South East
3	State Hospital, Ijebu-Ode, Ogun State	Urban	Secondary	South West
4	General Hospital, Alimosho, Lagos State	Urban	Secondary	South West
5	Oron General Hospital, Oron, Akwa Ibom State	Semi-urban	Secondary	South South
6	Calabar General Hospital, Calabar, Cross River State	Urban	Secondary	South South
7	Dalhatu Araf Specialist Hospital, Lafia, Nasarawa State	Semi-urban	Secondary	North Central
8	Faith Alive Foundation, Jos, Plateau State	Urban	Secondary	North Central
9	Gombe State Specialist Hospital, Gombe, Gombe State (GSSH)	Urban	Secondary	North East
10	General Hospital, Billiri, Gombe State	Urban	Secondary	North East
11	Dr. Gwamna Awan General Hospital, Kaduna, Kaduna State (GAGH)	Urban	Secondary	North West
12	General Hospital, Funtua, Katsina State	Semi-urban	Secondary	North West
