# Supplementary material for: Comparative effectiveness of implementation strategies for Accelerating Cervical Cancer Elimination through the integration of Screen-and-treat Services (ACCESS study): protocol for a cluster randomized hybrid type III trial in Nigeria
Source: Implement Sci. 2024 Mar 11;19:25. doi: 10.1186/s13012-024-01349-9 (PMC10926605; doi:10.1186/s13012-024-01349-9)
Supplement: Supplementary file 2 — Additional file 2. Measurement Instruments. [file 13012_2024_1349_MOESM2_ESM.pdf]

Additional file 2: Measurement Instruments

1. Organizational Readiness for Implementation Change (ORIC)

Health Facility: Dropdown menu

Name of Data collector (SRC): Dropdown menu

Date questionnaire was administered ………

Respondent's Designation: Dropdown

1	2	3	4	5
Disagree	Somewhat Disagree	Neither Agree nor Disagree	Somewhat Agree	Agree

1. People who work here feel confident that the organization can get people invested in implementing CCST	1	2	3	4	5
2. People who work here are committed to implementing CCST	1	2	3	4	5
3. People who work here feel confident that they can keep track of progress in implementing CCST	1	2	3	4	5
4. People who work here will do whatever it takes to implement CCST	1	2	3	4	5
5. People who work here feel confident that the organization can support people as they adjust to implementing CCST	1	2	3	4	5
6. People who work here want to implement CCST	1	2	3	4	5
7. People who work here feel confident that they can keep the momentum going in implementing CCST	1	2	3	4	5
8. People who work here feel confident that they can handle the challenges that might arise in implementing CCST	1	2	3	4	5
9. People who work here are determined to implement CCST	1	2	3	4	5
10. People who work here feel confident that they can coordinate tasks so that CCST implementation goesothly	1	2	3	4	5
11. People who work here are motivated to implement CCST	1	2	3	4	5
12. People who work here feel confident that they can manage the politics of implementing CCST	1	2	3	4	5

2. Implementation Climate Measure (ICM)

Health Facility: Dropdown menu

Name of Data collector (SRC): Dropdown menu

Date questionnaire was administered ………

Respondent's Designation: Dropdown

**Implementation Climate Measure – Unit Level (Health Facility)**					
	0	1	2	3	4
Please indicate the extent to which each statement applies to your health team.	Not at all	A little	Good	Very Good	A very great extent
1) Team members are expected to carry out CCST with a certain number of patients					
2) Team members are expected to help my health facility meet its goals for implementing CCST					
3) Team members get the support needed to identify potentially eligible patients for CCST					
4) Team members get the support needed to carry out CCST on patients					
5) Team members receive recognition when they carry out CCST on patients					
6) Team members receive appreciation when they use CCST with patients					

3. Measure of Innovation Specific Implementation Intentions (MISII)

Health Facility: Dropdown menu Name of Data collector (SRC): Dropdown menu

Date questionnaire was administered ……… Respondent's Designation: Dropdown

**Measure of Innovation Specific Implementation Intentions (MISII)**					
	0	1	2	3	4
Please answer the following questions about the extent to which you intend to use CCST	Not at all	To a slight extent	To a moderate extent	To a great extent	To a very great extent
1) I plan to carry out CCST with my eligible patients					
2) Using CCST is a high priority for me					
3) I will carry out the appropriate aspects of CCST with my patients					

4. CCST Self Efficacy: EBP Beliefs and Implementation Scales

Health Facility: Dropdown menu Name of Data collector (SRC): Dropdown menu

Date questionnaire was administered ……… Respondent's Designation: Dropdown

1	2	3	4	5
Strongly Disagree	Disagree	Neither Agree nor Disagree	Agree	Strongly Agree

	Please answer the following questions about implementation of cervical cancer screening and treatment (CCST)					
1.	I am sure that I can implement CCST in a time efficient way	1	2	3	4	5
2.	I am sure that I can implement CCST	1	2	3	4	5
3.	I am confident about my ability to implement CCST in my health facility	1	2	3	4	5
4.	I believe that I can overcome barriers in implementing CCST	1	2	3	4	5
5.	I know how to implement CCST sufficiently to make practice changes	1	2	3	4	5
6.	I can access the best resources in order to implement CCST	1	2	3	4	5
7.	I am clear about the steps of CCST	1	2	3	4	5

5. Provider REport of Sustainment Scale (PRESS)

Health Facility: Dropdown menu

Name of Data collector (SRC): Dropdown menu

Date questionnaire was administered ………

Respondent's Designation: Dropdown

Provider Report of Sustainment Scale (PRESS)					
	0	1	2	3	4
The following questions ask about CCST in your health facility.<br>***Please indicate the extent to which you agree with the following items***	Not at all	To a slight extent	To a moderat e extent	To a great exten t	To a very great extent
1) Staff carry out CCST as much as possible when appropriate					
2) Staff continue to carry out CCST throughout changing circumstances at this facility					
3) CCST is a routine part of our practice at this health facility					

6. Implementation Strategy Fidelity Checklist

Health Facility: Dropdown menu Name of Data collector (SRC): Dropdown menu , Date questionnaire was administered ……… Respondent's Designation: Dropdown

CORE IMPLEMENTATION FIDELITY TOOL (To be completed at 6 months, 12 months, and annually by the SRC /Administered on: Nurses, Doctors, and Adherence Counsellors)

Features	Adherence (Y=1 /N=0)	Exposure<br>1 – Never 2 –Rarely (less than 10% of chances in last 6 months) 3 – Sometimes (>10% but <70% chances in last 6mth)<br>4 – Always (≥70% of chances in the last 6 months)	
1. This facility participates in real-time consultation sessions using AVIVA	☐	**Period**: How often was the platform used in the last 6 months?<br>(a) Site Doctor ☐ Reason for exposure level: ____________<br>(b) Site Nurse ☐ Reason for exposure level: ____________	***SRC kindly document relevant information from Facility staff and Expert Reviewer in your field note***
2. This facility participates in periodic training sessions using AVIVA	☐	Period: How often was the periodic training session attended in the last 6 months?<br>(a) Site Doctor ☐ Reason for exposure level: ____________<br>(b) Site Nurse ☐ Reason for exposure level: ____________<br>**QUALITY:** On a scale of 1 to 3 (low to high quality), how well were the training/program components administered? ☐<br>**ENGAGEMENT:** On a scale of 1 to 3 (low to high engagement), how well did your facility staff actively participate? ☐	***SRC kindly document relevant information from Facility staff and Expert Reviewer in your field note***
3. This facility conducts educational meetings on CCST	☐	Period: How often was the educational meeting attended in the last 6 months?<br>(a) Site Doctor ☐ Reason for exposure level: ____________<br>(b) Site Nurse ☐ Reason for exposure level: ____________<br>(c) Administrator ☐ Reason for exposure level: ____________<br>**QUALITY:** On a scale of 1 to 3 (low to high quality), how well were the educational meeting components delivered? ☐<br>**ENGAGEMENT:** On a scale of 1 to 3 (low to high engagement), how well did your facility staff actively participate? ☐	***SRC kindly document relevant information from Facility staff in your field note***
4. This facility participates in stakeholders' meetings	☐	Period: How often was the periodic training session attended in the last 6 months?<br>(a) Site Doctor ☐ Reason for exposure level: ____________<br>(b) Site Nurse ☐ Reason for exposure level: ____________<br>(c)Administrator ☐ Reason for exposure level: ____________	***SRC kindly document any other relevant information on this strategy in your field note***

CORE IMPLEMENTATION FIDELITY TOOL (To be completed at 6 months, 12 months, and annually by the SRC /Administered on: Nurses, Doctors, and Adherence Counsellors)

5a. This facility has Functional Referral coordinators at (a) (this facility)	☐	**At this facility:** 1. RC fills referral form, 2. takes a picture of the referral form, 3. sends a picture of the referral form via WhatsApp to the receiving facility, 4. documents the referral in the referral register 5. gives the next appointment, 6. gives the original copy of the referral form to the patient **(as an example, If RC does all of the 6 steps, enter 6/6)**	☐
(b). Receiving facility	☐	What fraction of suspected cancer cases were all the 6 steps taken, in the past 6 months (report as fraction e.g 2/5), if none report 0.	☐

6. This facility provides: (Please enter Y for Yes or N for No) **(A) FLIERS** ☐ **(B) LEAFLETS** ☐ **(C) FLIP CHARTS** ☐<br><br>7. How often have these materials been displayed in the HIV clinic in the last 6 months? (Using the likert scale below, enter the appropriate number)<br>1 = NEVER 2= RARELY 3 = SOMETIMES 4 = OFTEN 5 = ALWAYS<br><br>**(A) FLIERS** ☐ **(B) LEAFLETS** ☐ **(C) FLIP CHARTS** ☐	***SRC kindly document the languages in which each of the three materials are displayed and any other relevant information in your field note***

CORE+ IMPLEMENTATION FIDELITY TOOL (To be completed at 6 months, 12 months, and annually by the SRC /Administered on: Nurses, Doctors, and Adherence Counsellors)

**Features**	**Adherence** (Y=1 /N=0)	**Exposure**<br>1 – Never 2 –Rarely (less than 10% of chances in last 6 months) 3 – Sometimes (>10% but <70% chances in last 6mth)<br>4 – Always (≥70% of chances in the last 6 months)
1. This facility participates in real-time consultation sessions using AVIVA	☐	**Period**: How often was the platform used in the last 6 months?<br>(a) Site Doctor ☐ Reason for exposure level: ______<br>(b) Site Nurse ☐ Reason for exposure level: ______<br>***SRC kindly document relevant information from Facility staff and Expert Reviewer in your field note***
2. This facility participates in periodic training sessions using AVIVA	☐	Period: How often was the periodic training session attended in the last 6 months?<br>(a) Site Doctor ☐ Reason for exposure level: ______<br>(b) Site Nurse ☐ Reason for exposure level: ______<br>***SRC kindly document relevant information from Facility staff and Expert Reviewer in your field note***<br>**QUALITY:** On a scale of 1 to 3 (low to high quality), how well were the training/program components administered? ☐<br>**ENGAGEMENT:** On a scale of 1 to 3 (low to high engagement), how well did your facility staff actively participate? ☐
3. This facility conducts educational meetings on CCST	☐	Period: How often was the educational meeting attended in the last 6 months?<br>(a) Site Doctor ☐ Reason for exposure level: ______<br>(b) Site Nurse ☐ Reason for exposure level: ______<br>(c) Administrator ☐ Reason for exposure level: ______<br>***SRC kindly document relevant information from Facility staff in your field note***<br>**QUALITY:** On a scale of 1 to 3 (low to high quality), how well were the educational meeting components delivered? ☐<br>**ENGAGEMENT:** On a scale of 1 to 3 (low to high engagement), how well did your facility staff actively participate? ☐
4. This facility participates in stakeholders' meetings	☐	Period: How often was the periodic training session attended in the last 6 months?<br>(a) Site Doctor ☐ Reason for exposure level: ______<br>(b) Site Nurse ☐ Reason for exposure level: ______<br>(c)Administrator ☐ Reason for exposure level: ______<br>***SRC kindly document any other relevant information on this strategy in your field note***
